# Editorial: Intercellular communication in chronic neuroinflammatory diseases

**DOI:** 10.3389/fncel.2026.1791491

**Published:** 2026-02-04

**Authors:** Maja Potokar, Jernej Jorgačevski, Dai Matsuse, Nunzio Vicario

**Affiliations:** 1 Laboratory of Neuroendocrinology - Molecular Cell Physiology, Faculty of Medicine, Institute of Pathophysiology, University of Ljubljana, Ljubljana, Slovenia; 2 Celica Biomedical, Ljubljana, Slovenia; 3 Department of Neurology, Graduate School of Medical Sciences, Neurological Institute, Kyushu University, Fukuoka, Japan; 4 Section of Physiology, Department of Biomedical and Biotechnological Sciences, University of Catania, Catania, Italy

**Keywords:** astrocytes, autophagy, neurodegeneration, neuroinflammation, radiotherapy, therapeutic strategies

Neurological conditions are a leading cause of disease burden worldwide (GBD 2021 Nervous System Disorders Collaborators, 2024), with millions of people affected by chronic inflammatory disorders of the central nervous system (CNS). Neuroinflammation, which follows trauma and infection of the CNS, is a common feature in the initiation and progression of neurodegenerative and demyelinating diseases such as Alzheimer's disease, Parkinson's disease, amyotrophic lateral sclerosis, multiple sclerosis, Huntington's disease, frontotemporal dementia, and neuromyelitis optica spectrum disorder. In view of recent progress in understanding inflammatory mechanisms in neurological disorders, this Research Topic, ‘*Intercellular Communication in Chronic Neuroinflammatory Diseases*', highlights diverse aspects contributing to chronic neuroinflammation and explores possibilities for therapeutic modulation. The compilation of review and original research manuscripts covers the contribution of different cell types in the CNS to neuroinflammation, with a focus on astrocytes and microglia. Intercellular communication among these cells and neurons occurs via the exchange of ions and small molecules mediated by channels and transporters in their plasma membranes, as well as through oxidative stress and degradation processes, such as autophagy. Aberrations in this communication is a common cause of chronic inflammation. Together, the contributions in this Research Topic provide complementary perspectives on the interplay of immune signaling, metabolic dysregulation, and glial biology, highlighting novel potential targets for diagnosis and intervention.

Among the mediators of neuroinflammation, connexins (Cxs) have emerged as a major focus of research in the field of neuroinflammation, with studies highlighting mechanistic insights and therapeutic possibilities for targeting Cxs. Cxs are a diverse family of transmembrane proteins that form hemichannels, enabling communication between intracellular and extracellular spaces and supporting homeostasis in the CNS (Vicario and Parenti, 2022). The review by Denaro et al. comprehensively covers the importance of Cxs in intercellular communication among CNS cells, particularly their function in glial intercellular communication and cell-induced inflammation, which is a process increasingly recognized as a unifying hallmark of various neurodegenerative diseases. As the predominant Cx in astrocytes and microglia, Cx43 is a central focus of this review, as this Cx has emerged at the forefront of ongoing basic and therapeutic research of neuroinflammation in diseases like Alzheimer's, Parkinson's, multiple sclerosis (MS), and Huntington's.

Intercellular communication that contributes to homeostasis and regulation of immune responses in the CNS occurs through various mechanisms. In addition to direct cell-to-cell contacts mediated by gap junction-forming Cxs, inflammatory responses are regulated by systemic immune modulation and by autocrine and paracrine signaling via cytokines, chemokines, growth factors, extracellular vesicles and lipid metabolites (Decrock et al., 2015). In their minireview, Müller and Di Benedetto focus on microglial crosstalk with neurons and astrocytes, placing microglia at the center of neuroimmune communication. Microglia-released cytokines and chemokines influence neuronal activity and the release of gliotransmitters and cytokines from astrocytes, and other microglia, affecting downstream steps of inflammatory cascades. The paper covers different molecular and cellular mediators of neuroinflammation and summarizes recent discoveries on the interaction between peripheral immunity and intercellular communication in the CNS. The authors also describe therapeutic strategies for neuroinflammation, along with the challenges that must be addressed to develop effective therapies that preserve protective immune functions and avoid immunosuppression.

Intercellular communication and inflammation are also affected by a number of intracellular processes that contribute to changes in the extracellular composition of nutrients, proteins, mitochondria, and extracellular vesicles. An example of such an intracellular process is autophagy, a conserved cellular degradation and recycling mechanism (Piletic et al., 2023). The manuscript by Potokar and Jorgačevski highlights the therapeutic potential of targeting autophagy and mitophagy in astrocytes, an area that has received considerably less attention than autophagy in neurons. Astrocytes are intricately involved in maintaining neuronal homeostasis and undergo phenotypic changes toward a reactive state during neurodegeneration. During inflammation, they release proinflammatory cytokines and, due to their strategic position at the blood-brain barrier, actively maintain its permeability, making them among the first CNS cells to encounter circulating drugs. The review summarizes possible modulators of autophagy and mitophagy, focusing only on those modulators that readily cross the blood-brain barrier.

Research that combines experiments on isolated cells and cell lines with animal models exhibiting signs and symptoms similar to human neurodegenerative diseases is essential for understanding intercellular communication and inflammation in the nervous system. Selecting an appropriate model must be closely aligned with the pathophysiology of the disease or biological process under investigation. Hashimoto et al. summarize the diversity of models used in studies of neurological disorders. Mouse models are the most widely used in biomedical research, as they share a high degree of genetic and physiological similarity with humans. In addition, zebrafish models are increasingly used, as certain features make zebrafish critical for investigating neurodegenerative diseases and potential treatment strategies. Comparative studies using these models can help identify candidate molecular factors with therapeutic relevance for promoting neural regeneration in mammalian neurological disorders.

Torrisi et al. review the regulation of neural stem cells (NSCs) fate in the context of redox homeostasis, focusing on the crosstalk between neuroinflammatory microenvironment and redox-sensitive signaling pathways. As a unique reservoir of plasticity within the largely non-regenerative CNS, NSCs operate at the dynamic interface between neurogenesis, tissue repair and the pathophysiological stresses imposed by chronic neuroinflammation. A recurring feature of neuroinflammatory environments is oxidative stress, which reshapes key morphogenic pathways and influences NSCs fate and proliferation. The review highlights the NRF2/Keap1 pathway as a master regulator of the NSC stress responses and explores its interplay pathways governing NSCs proliferation and differentiation, including the NRF2–Notch axis. Finally, the authors discuss how mitochondrial dynamics and metabolic reprogramming, particularly the transition from glycolytic to oxidative metabolism, modulate NSCs function during chronic neuroinflammation.

This Research Topic also includes two original research papers. Ali et al. investigate environmental factors, such as chronic ethanol use, that cause glia-mediated neuroinflammation and oxidative stress, ultimately impairing brain function. Using a mouse model, they demonstrate activation of astrocytes and microglia, accompanied by increased expression of pattern recognition receptors and kinases associated with neuroinflammatory responses due to prolonged ethanol exposure. Moreover, the authors report the neuroprotective potential of saikosaponin-A (SSA) in ethanol-induced neurodegeneration, which was reflected in improved synaptic plasticity and cognition.

The paper by Palazzo et al. presents innovative research on chronic inflammation in relapsing-remitting MS (RR-MS). MS is the most common chronic inflammatory neurodegenerative disease of the CNS, in which immune-mediated myelin damage leads to axonal degeneration in the brain and spinal cord (Dendrou et al., 2015). Beyond the current diagnostic approaches, which rely primarily on clinical and radiological criteria, the authors aimed to identify new biomarkers of RR-MS. To this end, they focused on extracellular vesicles, specifically exosomes, which are known to modulate inflammatory responses in the CNS and have emerged as promising biomarkers and therapeutic targets in MS (Hasaniani et al., 2024). Their research is intriguing as it reveals altered lipid signatures in circulating exosomes of RR-MS patients and paves the way for further insights into disease mechanisms, as well as the development of diagnostic tools and therapeutic applications.

Summarized, this Research Topic brings together complementary perspectives, shedding light on the complex interplay between intercellular communication and chronic neuroinflammation. A central theme across the contributions is the active role of glial cells, particularly astrocytes and microglia, in shaping immune responses within the CNS. Collectively, the included reviews and original research manuscripts advance our understanding of neuroinflammatory mechanisms and highlight translational opportunities for modulating inflammation and developing diagnostic tools for neurodegenerative diseases.
